# Treatment of Direct Blending Dye Wastewater and Recycling of Dye Sludge 

**DOI:** 10.3390/molecules17032784

**Published:** 2012-03-06

**Authors:** Xin-Hui Xu, Ming-Li Li, Yuan Yuan

**Affiliations:** State Key Laboratory of Pollution Control and Resource Reuse, College of Environmental Science and Engineering, Tongji University, Shanghai 200092, China; Email: xinfei0105@163.com (X.-H.X.); Enviklab@tongji.edu.cn (Y.Y.)

**Keywords:** barium sulfate, direct blending dye, hybrid material, dye wastewater treatment, reuse of sludge

## Abstract

A new sorbent material, barium sulfate-Direct Blending Yellow D-3RNL hybrid (BSD), was synthesized and characterized by various methods. Both the anionic dyes, Reactive Brilliant Red X-3B and Weak Acid Green GS were hardly adsorbed by the BSD material, while the sorption of Ethyl Violet (EV) and Victoria Blue B were extremely obvious. The sorption of cationic dyes obeyed the Langmuir isotherm model, which depended on the electric charge attraction. The saturation amount of EV adsorbed onto the BSD material approached to 39.36 mg/g. The sorption of EV changed little with pH from 3 to 12 while it increased with increasing levels of electrolyte. A dye wastewater sampled from Jinjiang Chemicals was treated, and the color removal rate was more than the COD removal rate. In addition, the cationic dye-BSD sludge was utilized as a colorant fill-in coating. The light stability and thermal stability of the colorant was measured and exhibited good features. This work provided a simple and eco-friendly method for dye wastewater treatment with recycling of waste.

## 1. Introduction

A growing population leads to rapid proliferation of industries and their pollution. Over 10,000 dyes and more than 700,000 tons of dyes are produced every year, and over 5% are discharged into the aquatic environment [1]. Many dyes are discharged into water as a waste and this causes very serious pollution as they are difficult to degrade [2,3]. Most dyes contain azo groups or aromatic rings, which are mutagenic and carcinogenic [4]. In recent years, the Yangtze Delta Area of China has been suffering from serious dye wastewater pollution, which causes damage to people’s health [5]. Therefore, dye wastewater pollution is a very important issue that needs to be solved.

In recent years physical or chemical treatment processes have been developed to treat dye wastewater, such as adsorption, biological treatments, electrochemical, flocculation–coagulation, advanced oxidation processes and membrane separation [6,7,8,9,10,11,12]. Some of them have proved effective, but they are often expensive and complex. An economical and easy solution to the treatment of highly concentrated dye wastewater is still an important problem faced by the dye industries. Adsorption is considered to be a simple and effective technology with wide potential applications in dye wastewater treatment. Many adsorbents for dye removal, such as activated carbon, agricultural by-products, fly ash, sewage sludge, clays and zeolites have been investigated [13,14,15,16,17,18]. However, most of the absorbents have low adsorption capacity, slow adsorption equilibria and are expensive, and the sludge produced in the treatment is another pollution problem.

**Figure 1:**
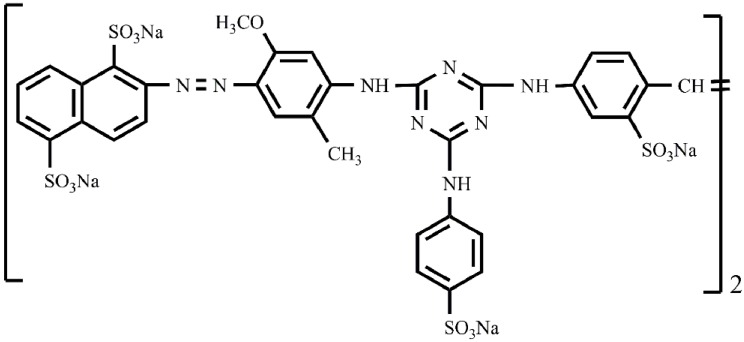
The chemical structure of direct blending yellow D-3RNL.

The concept of inorganic/organic hybridization is applied extensively in composite materials, which are used as biomaterials, catalysts, thin-films, photosensitive cells, *etc*. [19,20,21]. However, this method is seldom considered in wastewater pollution control. Recently, Gao’s group have described some hybrids used in dye wastewater treatment, for example, a Ag(SCN)-tetrabromo/tetrachlorofluorescein hybrid material [22], calcium oxalate-Bromopyrogallol Red hybrid material [23], and calcium carbonate-Weak Acidic Pink Red B hybrid material [24]. Zhao and Gao [25] developed a facile treatment of a dye wastewater mixture by *in situ* hybridization with growing calcium carbonate. Oladoja [26] also reported a method similar to Zhao and Gao’s method. This work has developed a low-cost sorbent by hybridizing Direct Blending Yellow D-3RNL with barium sulfate. As a conventional dye, D-3RNL (Figure 1), with its eight sulfonic groups, exhibits a good nucleophilicity with alkaline-earth metals, e.g., Ba^2+^. 

Barium sulfate is often used as additive in coatings, plastics, and paper to improve their toughness and impact strength [27,28]. Thus, barium sulfate and D-3RNL were selected for synthesizing a conjugate–BaSO_4_ hybrid to treat dye wastewater and the dye-BSD sludge produced during the treatment process may have the potential to be reused as colorant fill-in coating. Thus, the dye wastewater and sludge will be treated simultaneously to decrease the discharge of organic pollutants to the environment.

## 2. Results and Discussion

### 2.1. Preparation and Characterization of the Dye Conjugate–BaSO_4_ Hybrid

**Figure 2:**
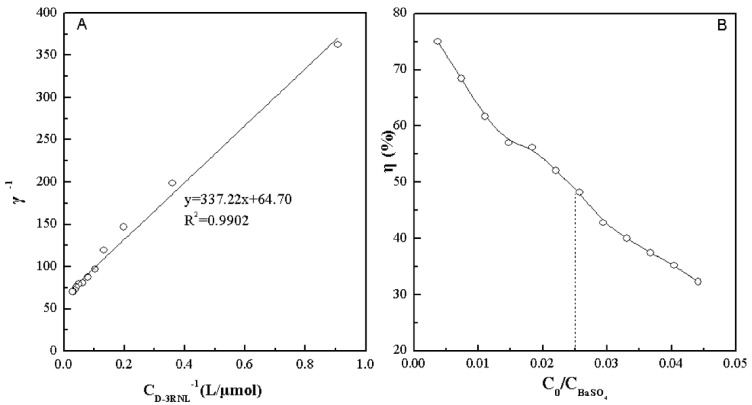
Effects of D-3RNL on its binding mole number (**A**) and reaction rate η (**B**). c_0_, and c _D-3RNL_ are the initial and equilibrium molarity, c_BaSO4_ is the initial molarity of SO_4_^2−^ and γ is the mole ratio of D-3RNL to BaSO_4_ in the BSD hybrid.

The hybridization of D-3RNL into BaSO_4_ obeyed the Langmuir sorption isotherm when freshly formed (Figure 2A). The saturation mole number (*N*) of D-3RNL to BaSO_4_ was calculated to be 1/64.7 and the binding constant (*K*) was 1.92 × 10^5^ M^−1^. The reaction rate (*η*) of D-3RNL is less than 50% when the initial mole ratio of D-3RNL to BaSO_4_ is more than 0.025 (Figure 2B). Referring to the previous research [24,29] and considering the reaction rate of D-3RNL, the D-3RNL conjugate–BaSO_4_ hybrid material (BSD) was prepared with the molar ratio for the dosage of Ba^2+^, SO_4_^2−^ and D-3RNL is 1.5:1:0.025. The material was dried and determined to be C 2.635%, N 0.819% and H 0.516% by elemental analysis. Thus, the molar ratio of D-3RNL to BaSO_4_ was calculated to be about 1/124 in the hybrid.

**Figure 3:**
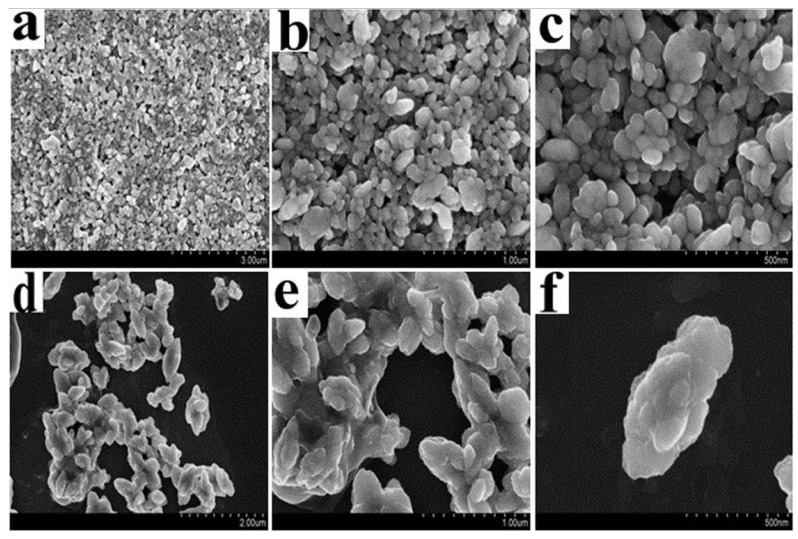
SEM images of the materials. a,b,c—BaSO_4_-only and d,e,f—BaSO_4_–D3RNL hybrid.

D-3RNL played an important role in control of the size and morphology of the hybrid. From the SEM images, the BaSO_4_-only particles look like pebbles and their surface is smooth (Figure 3a–c). However, the BSD particles look discoidal and have rough surfaces with many small globes embedded into the particles (Figure 3d–f). Besides, the addition of D-3RNL affected the size of the particles. The size of the BSD particles was bigger than the BaSO_4_-only, which would be beneficial for the sedimentation of the dye-BSD sludge.

**Figure 4:**
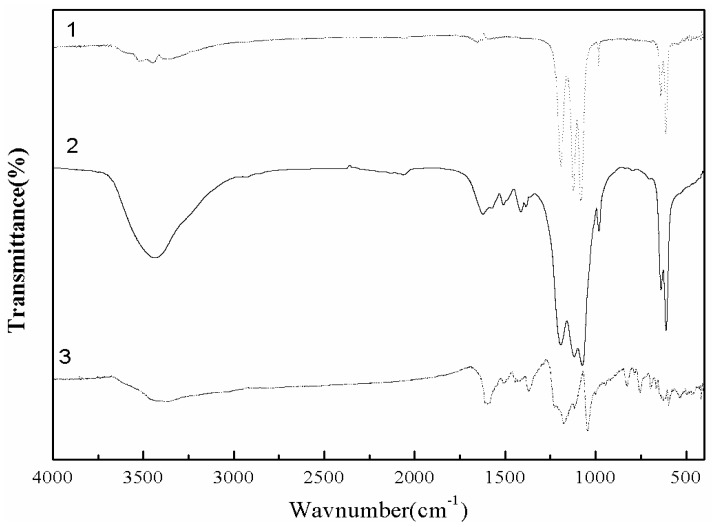
FTIR of BaSO_4_ (**1**), BaSO_4_–D3RNL hybrid (**2**), D3RNL (**3**).

The FTIR spectra (Figure 4) revealed the difference in composition between the BaSO_4_-only and the BSD material. It is well-known that BaSO_4_ has characteristic vibration bands, such as the peaks near 1195, 1119 and 1075 cm^−1^ corresponding to the sulfate absorption bands [30]. Both curves have the characteristic vibration bands of sulfate, but compared to unmodified BaSO_4_ (Figure 4, curve 1), curve 2 shows that the FTIR spectrum of the BSD material was more complicated. In the FTIR of the BSD material, all the characteristic bands corresponding to D-3RNL and BaSO_4_ appear on the right positions. The characteristic bands of BSD appear at 3440, 1623, 1509, 1413, and 1385 cm^−1^ due to the N–H stretching vibrations, the C=C vibration band of benzene ring (1623, 1509 and 1413 cm^−1^), and the C-H band of benzene ring (1385 cm^−1^), which prove the D-3RNL conjugate hybridization with the growing barium sulfate. 

The ζ-potential of the BSD material was measured to be −21.13 mV, while BaSO_4_-only material had +1.11 mV of ζ-potential. This means the BSD material would form a negatively electronic aggregate in aqueous media [24]. From the SEM, FTIR and ζ-potential research, it could be concluded that D-3RNL was absorbed and occluded in BaSO_4_ particles during the co-precipitation process. A dye conjugate–BaSO_4_ hybrid formed, which was negatively charged due to the sulfonic acid groups (Figure 1).

### 2.2. Sorption Selectivity

**Figure 5:**
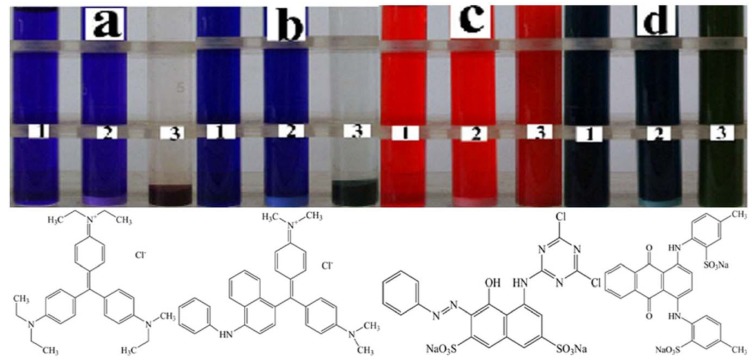
Photos illustrating color change of dye solutions and their structures: EV (**a**), VBB (**b**), RBRX-3B (**c**), WAGGS (**d**). All the dyes were added 200 μmol/L. All liquids were settled for 10 min. 1 Dye-only. 2 treated with 0.18% BaSO_4_-only, 3 treated with 0.18% BSD material.

In order to investigate the sorption selectivity and capacity of the BSD material, the two cationic dyes e.g., EV (**a**) and VBB (**b**), and two anionic dyes e.g., RBRX-3B (**c**) and WAGGS (**d**) were tested. From the photos in Figure 5, the color removal of EV (**a**) and VBB (**b**) were extremely obvious and the sediment turned green for VBB and brown for EV. 

The BSD material had little effect to both the anionic dyes. The BaSO_4_-only material didn’t show any obvious removal of any of the dyes. The sorption selectivity clarified that the interaction of cationic dye with the material was mainly due to the charge attraction. The cationic dyes are positively charged (Figure 5a,b) and they could be quickly absorbed to the BSD hybrid. On the contrary, the anionic dyes were excluded by the BSD hybrid due to their negative charges (Figure 5c,d). Thus, the color didn’t change much.

### 2.3. Sorption Isotherm

**Figure 6:**
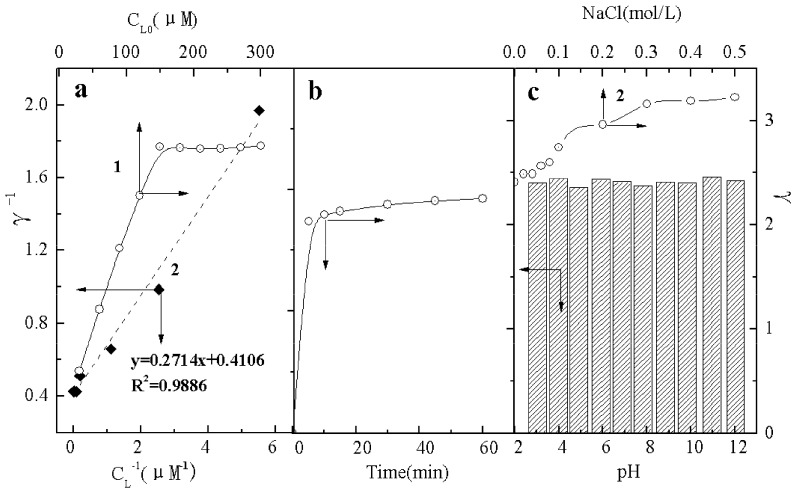
(**a**) Effect of EV (C_L0_). 1- change of the mole ratio (γ) of EV onto BSD (0.18%) and 2- plots γ^−1^ *vs.* C_L_^−1^; (**b**) Effect of time: variation of the mole ratio (γ) of EV onto BSD (0.18%), C_L_ = 150 μmol/L, pH = 7; (**c**) Effects of pH (column) and electrolyte (curve): variation of mole ratio (γ) of EV onto BSD (0.18%), C_L_ = 150 μmol/L.

A cationic dye e.g., EV was selected to investigate the sorption performance and isotherm. Figure 6a shows the sorption isotherm of EV onto the BSD material. The sorption amount of EV increased with increase of the initial concentration of EV (*C_L0_*) and then approached saturation when *C_L0_* was more than 150 μmol/L. The sorption of EV fits the Langmuir isotherms model, which indicated the monolayer sorption. The saturation molar number (*N*) of EV was calculated to be 2.45 mol EV/mol D-3RNL, *i.e*., 39.36 mg EV/g (the BSD material) and the sorption constant (*K*) was calculated to be 1.51 ×10^6^ M^−1^. Therefore, the sorption of cationic dye was sufficient and stable on the BSD material. This will be beneficial for the reuse of dye sludge.

The sorption of EV was almost complete in 5 min (Figure 6b) while it took over 30 min onto activated carbon [24]. The pH from 3 to 12 hardly affected on the sorption resulting from the relatively stable performance of the BSD material (Figure 6c). The electrolyte concentration had an obvious effect on the sorption (Figure 6c2). The γ of cationic dye increased with increased electrolyte, which is beneficial for treatment of a concentrated salt dye wastewater. 

### 2.4. Treatment of Dye Wastewater

**Figure 7:**
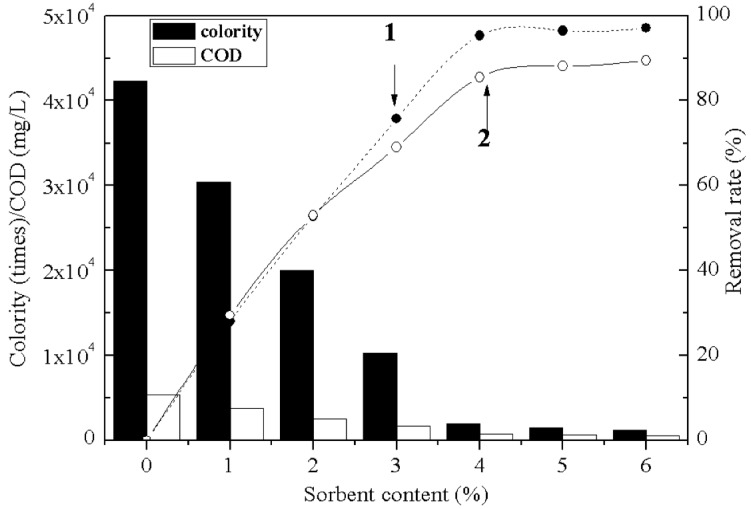
Changes of colority/COD and their removal rate (1, 2) in the cationic dye wastewater when treated with the BSD material added from 1% to 6%.

With increasing public awareness of environmental protection, the recycling and reuse of waste has long been studied in order to reduce the emission of pollutants and treatment costs [31,32]. The BSD material was prepared with the D-3RNL dye wastewater discharged from dye plant instead of the reagent, and it was applied as the sorbent to treat cationic dye wastewater. Figure 7 shows the colority and COD decrease with increase of the sorbent. The colority of the dye wastewater decreased to 2,000 from 43,000 and COD to 800 mg/L from 5,400 mg/L when 4% of the sorbent was added. The COD removal rate was less than the colority removal rate. It indicated that not all the organic matters had been captured by the BSD material, e.g. small uncharged organic molecules. The colority and COD decreased obviously when the sorbent was less than 3%. The BSD material was suggested treating the high concentration dye wastewater. 

### 2.5. Recycling of the Dye Sludge

**Figure 8:**
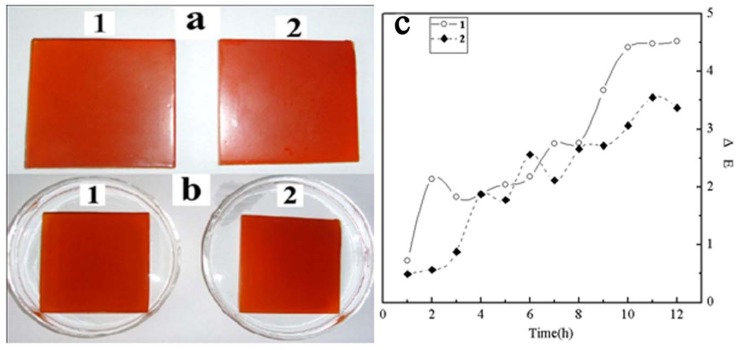
Photos (**a**,**b**) illustrating the color change of the colored coating. (**a**) the colored coating; (**b**) the colored coating immersing in acidic (1) and basic (2) aqueous media; (**c**) ΔE change with time, curve 1- heated at 100 °C and 2- irradiated with a UV light at 1000 W.

As is well known, barium sulfate is often used as additive filler in coatings, so the dye-contaminated sludge produced from the wastewater treatment could be tried as color filler for coatings (Figure 8a). As an example, a colored coating was prepared by adding the above sludge into a mixture of waterborne epoxy coating and curing agent, and then was brushed onto the glass. The colored sludge was found to be dispersed in the coating without sediment and in Figure 8a it shows uniform color. The plate-shaped product was immersed in acidic (1 mol/L HCl, Figure 8b1) and basic (1 mol/L NaOH, Figure 8b2) media. No color substance was found to release after 24 h immersion. The color of the coating filled with the sludge exhibited a small change with ΔE values (total color change values) of 3.1 and 4.2 for acidic and basic media immersion, suggesting that the two media have little influence on the coating. In order to investigate the color stability of sludge, its light stability and thermal stability have been studied. From Figure 8c, the ΔE increased with an increase in heating time and UV irradiation time. The ΔE was less than 5 after heating for 12 h (Figure 8c1 ), and less than 4 after UV irradiation for 12 h (Figure 8c2 ). The dye-contaminated sludge was proved to be light and thermally stable in the waterborne epoxy coating. Thus, the dye-contaminated sludge has the potential to be used as colorant for some coatings. Compared to incineration or landfill, the dye sludge can be recycled and the secondary pollution could be reduced.

## 3. Experimental

### 3.1. Apparatus and Materials

A Model S4100 photodiode array spectrometer (Scinco, Korea) was used to determine the colority and concentration of color compounds and the suspended substance liquids. A Model TG16-WS centrifuger (Hunan Xiangyi Instruments, China) was used to separate the hybrid materials from liquid. A Model Quanta 200 FEG scanning electronic microscope (SEM, FEI Co., USA) was used to measure the size and shape of the materials. Infrared (IR) spectra of the hybrid in KBr pellets were obtained, using an Equinoxss/hyperion 2000 infrared spectrometer system (BRUKER Co., Germany). A Model Vario EL III elemental analyzer was used to analyze the elemental composition of the materials. A Model 5B-1 programmable digestion system (Lanzhou, China) and a Model PORS-15V portable chemical oxygen demand (COD) analyzer (Pgeneral Co., China) were coupled for determination of COD in wastewater. A Model/WSC-Y colorimeter (BOIF, China) was used to determine the color factors (L, a and b) of color coating. The following solutions were prepared by dissolving in deionized water: Direct Blending Yellow D-3RNL (C.I. 161, Tongluo Chemicals Co., Ltd (Suzhou, China), 45%), Victoria Blue B (VBB, C.I. 44045, Aladdin Co., 80%), Ethyl Violet (EV, C.I. 42600, Aladdin Co., 90%), Reactive Brilliant Red X-3B (RBRX-3B, C.I.18200, Shuang Hong Chemicals Co., Ltd (JinHua, China), 75%), Weak Acid Green GS (WAGGS, C.I.61570, Aladdin Co., 95%), Na_2_SO_4_ (0.02 mol/L, AR, Aladdin Co.) and BaCl_2_ (0.02 mol/L, AR, Aladdin Co.). A cationic dye wastewater was sampled from Jinjiang Chemicals (Hangzhou, China) and its colority determined to be 43,000 (yellow) and COD 5400 mg/L. The waterborne epoxy coating and curing agent (CV-600) was purchased from Fulang Chemicals Co., Ltd (Shanghai, China).

### 3.2. Preparation and Characterization of the Dye Conjugate–BaSO_4_ Hybrid

Direct Blending Yellow D-3RNL (100 mL, 2 mmol/L) was mixed with sodium sulfate (400 mL, 0.02 mol/L) and mixed thoroughly, then barium chloride (600 mL, 0.02 mol/L) was added slowly under stirring. After 30 min, the suspending substance product was precipitated and washed with deionized water (1,500 mL) three times. Finally, approximately 18% (W/V) product aqueous liquid was prepared with deionized water. The surface electricity of the suspending sorbent in liquid was measured by a ζ-potential detecting-device. The elemental analysis, SEM and FTIR of the BSD material powders were measured.

### 3.3 Sorption of Dyes

Four dyes (200 μmol/L), RBRX-3B, WAGGS, EV, and VBB were prepared for investigating the sorption selectivity, performance, and mechanism of the BSD material. The concentration of dyes was determined by spectrophotometry. 

### 3.4. Treatment of Dye Wastewater

The cationic dye wastewater sampled from Jinjiang Chemicals (colority 43,000 times and COD 5400 mg/L) was mixed thoroughly with the BSD suspending substance liquid. After 30 min, the colority of supernatant was determined by spectrophotometry. The unit of “times” means that the dilution times when the wastewater was diluted until the absorbance is lower than 0.005, which has the same absorbance with distilled water. The COD was determined by the COD analyzer. Thus, the colority removal rate of dye wastewater and the removal rate of COD were calculated. 

### 3.5. Reusing Dye-Contaminated Sludge as Colorants

The dye-BSD sludge formed above was separated from the treated wastewater and then concentrated. 10 mL (75%, W/V) of the concentrated sludge was mixed together with 100 g of waterborne epoxy coating and 30 g curing agent. After mixing thoroughly, it was brushed on glasses (60 mm × 60 mm × 3 mm) and dried at room temperature for 7 days. Then, the glass brushed with colored coating was immersed in 1 mol/L HCl aqueous solution and 1 mol/L NaOH aqueous solution for 24 h, respectively, where both the solutions were 30 mL in volume. In order to investigate light stability and thermal stability of the colored coating, the prepared samples was irradiated by an ultraviolet lamp (1,000 W) for 12 h and heated at 100 °C for 12 h, respectively. The release amount of colored substances and the color change of the samples were observed before and after irradiation, heating and immersing. The *L*, *a*, and *b* values of color were determined according to the CIE-lab standard and the change in color, expressed as Δ*E*, was calculated. During measuring of the *L*, *a* and *b* values, a couple of sheets of white printing paper were placed below the colored paint samples. The Δ*E* was calculated by the following equation [33]:



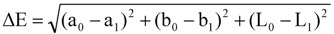



where *L_0_*, *a_0_* and *b_0_* was the initial color values of the colored coating; *L_1_*, *a_1_* and *b_1_* was the color values after irradiation, heating, and immersing.

In the CIELAB color system, the absolute magnitude of color change between two conditions is given by Δ*E*. A Δ*E* value of one unit is approximately equivalent to a color difference that is just visually perceptible to 50% of observers under controllable conditions. Value of Δ*E* from 2 to 3 represents the color difference that is slightly perceptible, and more than 3.3 is visually perceptible to 50% of observers [34]. Δ*E* more than 7 shows a marked color difference [35]. 

## 4. Conclusions

A novel sorbent was prepared by immobilizing D-3RNL into growing BaSO_4_, and the sorption of EV was investigated. The sorption of the cationic dye caused by the charge attraction fits the Langmuir isotherm model. The effects of pH, electrolyte, and sorption time were investigated and the sorption is fast, affected only a little by pH and favorable for a high salt dye wastewater. The BSD material was used to treat a cationic wastewater with satisfactory results. The dye-BSD sludge was filled in a waterborne epoxy coating. The color of the colored paint was stable when immersed in acidic and basic media and changed little when irradiated by a UV lamp. This work provided an eco-friendly and facile method for treatment of dye wastewaters.
